# A specific gut microbiota signature is associated with an enhanced GLP-1 and GLP-2 secretion and improved metabolic control in patients with type 2 diabetes after metabolic Roux-en-Y gastric bypass

**DOI:** 10.3389/fendo.2023.1181744

**Published:** 2023-10-17

**Authors:** Laura Hernández-Montoliu, M-Mar Rodríguez-Peña, Rocío Puig, Brenno Astiarraga, Fernando Guerrero-Pérez, Nuria Virgili, Rafael López-Urdiales, Javier Osorio, Rosa Monseny, Claudio Lazzara, Lucía Sobrino, Manuel Pérez-Maraver, María Pérez-Prieto, Silvia Pellitero, Sonia Fernández-Veledo, Joan Vendrell, Nuria Vilarrasa

**Affiliations:** ^1^ Department of Endocrinology and Nutrition, Bellvitge University Hospital-Institut d'Investigació Biomédica de Bellvitge, L’Hospitalet de Llobregat, Barcelona, Spain; ^2^ Hospital Universitari Joan XXIII de Tarragona, Institut d’Investigació Sanitària Pere Virgili (IISPV), Tarragona, Spain; ^3^ CIBER de Diabetes y Enfermedades Metabólicas Asociadas (CIBERDEM)-Instituto de Salud Carlos III (ISCIII), Madrid, Spain; ^4^ Department of Endocrinology and Nutrition Hospital de la Santa Creu i Sant Pau, Institut de Recerca de l’Hospital de la Santa Creu i Sant Pau, Barcelona, Spain; ^5^ Department of Medicine, Universitat Autònoma de Barcelona, Barcelona, Spain; ^6^ Department of General and Gastrointestinal Surgery. Bellvitge University Hospital-Institut d'Investigació Biomédica de Bellvitge, L’Hospitalet de Llobregat, Barcelona, Spain; ^7^ Clinical Nutrition Unit. Bellvitge University Hospital-Institut d'Investigació Biomédica de Bellvitge, L’Hospitalet de Llobregat, Barcelona, Spain; ^8^ Department of Endocrinology and Nutrition and Health Sciences Research Institute and University Hospital Germans Trias i Pujol, Badalona, Spain; ^9^ Department of Medicine and Surgery, Universitat Rovira i Virgili (URV), Reus, Spain

**Keywords:** incretin, microbiota, type 2 diabetes remission, severe obesity, bariatric surgery

## Abstract

**Objective:**

To determine changes in incretins, systemic inflammation, intestinal permeability and microbiome modifications 12 months after metabolic RYGB (mRYGB) in patients with type 2 diabetes (T2D) and their relationship with metabolic improvement.

**Materials and methods:**

Prospective single-center non-randomized controlled study, including patients with class II-III obesity and T2D undergoing mRYGB. At baseline and one year after surgery we performed body composition measurements, biochemical analysis, a meal tolerance test (MTT) and lipid test (LT) with determination of the area under the curve (AUC) for insulin, C-peptide, GLP-1, GLP-2, and fasting determinations of succinate, zonulin, IL-6 and study of gut microbiota.

**Results:**

Thirteen patients aged 52.6 ± 6.5 years, BMI 39.3 ± 1.4 kg/m^2^, HbA_1c_ 7.62 ± 1.5% were evaluated. After mRYGB, zonulin decreased and an increase in AUC after MTT was observed for GLP-1 (pre 9371 ± 5973 vs post 15788 ± 8021 pM, *P*<0.05), GLP-2 (pre 732 ± 182 vs post 1190 ± 447 ng/ml, *P*<0.001) and C- peptide, as well as after LT. Species belonging to Streptococaceae, Akkermansiacea, Rickenellaceae, Sutterellaceae, Enterobacteriaceae, Oscillospiraceae, Veillonellaceae, Enterobacterales_uc, and Fusobacteriaceae families increased after intervention and correlated positively with AUC of GLP-1 and GLP-2, and negatively with glucose, HbA_1c_, triglycerides and adiposity markers. *Clostridium perfringens* and *Roseburia* sp. *40_7* behaved similarly. In contrast, some species belonging to Lachnospiraceae, Erysipelotricaceae, and Rumnicocaceae families decreased and showed opposite correlations. Higher initial C-peptide was the only predictor for T2D remission, which was achieved in 69% of patients.

**Conclusions:**

Patients with obesity and T2D submitted to mRYGB show an enhanced incretin response, a reduced gut permeability and a metabolic improvement, associated with a specific microbiota signature.

## 1 Introduction

Bariatric surgery (BS) is a highly effective therapy for patients with obesity and type 2 diabetes mellitus (T2D), and many mechanisms have been proposed for its metabolic benefits (1). One of the main drivers of T2D improvement is the enhanced delivery of nutrients and bile to the distal gastrointestinal (GI) tract as a consequence of anatomical rearrangement, along with a rapid gastric emptying leading to increased nutrient-stimulated secretion of such gut hormones as glucagon-like peptide 1 (GLP-1), peptide YY and oxyntomodulin, implicated in the improvement of βcell function and food intake regulation (2) (3) (4). Also, caloric restriction, weight loss, reduction in insulin resistance (IR) and decreased pancreatic and hepatic fat deposits have been implicated in T2D remission. More recently, bile acid diversion and gut microbiome have been recognized as important factors in the complex network of glucose homeostasis after BS (5) (6) (7) (8) (9). Although current knowledge links gut microbiota to host glucose metabolism, the mechanisms are still unclear (10) (11).

Low concentrations of Firmicutes and an increase in the relative abundance of Gammaproteobacteria and *Akkermansia muciniphila* after Roux-en-Y gastric bypass (RYGB) have been observed among humans (12) (13) (14) (15) and rodents (16) (17) (18). These changes seem to contribute to decreased intestinal permeability, improving IR. Few previous studies have characterized the microbiota directly linked to metabolic improvement and T2D remission after RYGB, although findings have been heterogeneous. In some, a more significant number of Actinobacteria was observed (19). In others, there was a higher abundance of *Eubateriaceae* and *Alistipes putredinis* pre-surgery, and *Lachnospiraceae* and *Roseburia* 12 months after surgery, in patients achieving T2D remission (20). However, studies analyzing the direct relationship between incretin secretion and specific gut microbiota species are scarce in human subjects with obesity (21) and absent in subjects with severe obesity and T2D.

In this scenario, we aimed to determine the changes in enteroendocrine hormones, systemic inflammation, intestinal permeability and microbiome modifications 12 months after metabolic RYGB (mRYGB) in patients with obesity and T2D. Also, we studied the relationship between these changes and the metabolic improvement. The follow-up time was set at 12 months since this is the point where the weight reaches its nadir after bariatric surgery and then stabilizes (22). For this reason, we consider that it is the best time to study changes in body composition, metabolism, and microbiota.

## 2 Materials and methods

A prospective single-center, non-blinded non-randomized controlled trial study was conducted, including patients with classes II and III obesity and T2D undergoing mRYGB. Patients were consecutively recruited from the obesity outpatient clinic of Bellvitge University Hospital. The inclusion criteria were as follows: age between 18 and 60 years old, body mass index (BMI) 35–43 kg/m^2^, T2D on hypoglycemic agents, insulin, or both. The exclusion criteria were the following: type 1 diabetes or positivity for glutamic acid decarboxylase autoantibodies, secondary forms of diabetes, acute metabolic complications in the previous 6 months, liver disease, renal dysfunction, previous BS, pregnancy, breastfeeding, or desired pregnancy in the 12 months following inclusion, and corticoid use by oral or intravenous route for more than 14 consecutive days in the previous three months. All patients signed informed consent, the protocol study (PI14/01997) was approved by the Clinical Research Ethics Committee (reference PR 198/14) and conducted in according with the principles of the Declaration of Helsinki.

At baseline and one year after surgery, patients underwent an anthropometric and body composition analysis with DEXA (HoQDR 4500; Hologic Inc., Waltham, MA), a complete biochemical examination, a standardized meal tolerance test (MTT), and a lipid test (LT). MTT consisted in the intake of 200ml of a standard meal (16% proteins, 49% carbohydrates, and 30% lipids [320 kcal]; Isosource Energy^®^, Nestle Health Science) over 5 min. Blood was sampled before meal ingestion (time 0 min) and at 15, 30, 60, and 120 min after meal ingestion. The LT was performed using an oral lipid solution ingested over 5 min, containing 50 g of fat in 100 mL of solution, of which 30% was saturated, 49% was monounsaturated, and 21% was polyunsaturated. Blood samples were drawn at fasting state (time 0 min) and at 60, 120, and 180 min after lipid ingestion.

All pharmacological treatment was stopped three days before the functional tests, except insulin treatment which was stopped 12 hours before the tests. Proton pump inhibitors were prescribed after surgery for only three months. In the month preceding the surgical procedure, all patients adhered to a very low-calorie diet, characterized by an intake of <800 kcal/day. This dietary regimen was achieved through the implementation of meal replacement with Optifast^®^ Nestle HealthScience, which is composed of 0.7 kcal/ml with a macronutrient distribution of 40% proteins, 30% carbohydrates, and 30% fats. Following the surgical intervention, diligent oversight was maintained over all patients by licensed dietitians, who provided guidance on adhering to a well-balanced diet, inclusive of all essential nutrients as stipulated by clinical guidelines for gastric bypass surgery (23). Probiotics were not administered to any of the patients to prevent potential interference with the microbiota analysis outcomes.

During the MTT and LT, plasma, insulin, C-peptide, GLP-1 and GLP-2 were determined at all-time points, whereas succinate, IL-6 and zonulin concentrations were only determined in the fasting state. Plasma insulin and C-peptide were determined by immunochemiluminometric assay (ADVIA Centaur, Siemens Healthcare, Erlangen, Germany), total plasma GLP-1 and GLP-2 by ELISA technique (respectively, EZGLP1T-36K and EZGLP-2-37K, Merck KGaA, Darmstadt, Germany, respectively) and plasma succinate on plasma filtrate (10.000 kD) using a fluorometric assay (EnzyChromTM Succinate Assay Kit, BioAssay Systems; USA). Fasting IL-6 and zonulin were analyzed by using ELISA high sensitivity kit (HS600C, USA R&D Systems, Inc., Minneapolis, MN) and ELISA (K5601, Immundiagnostik AG, Bensheim, German), respectively. Glucose and cholesterol were quantified using molecular absorption spectrometry, employing the Cobas^®^ 8000 system by Roche Diagnostics. Specifically, glucose levels were determined using the GLUC3 Gen.3 assay on the cobas c503 platform (Roche Diagnostics, reference number 08057800190), while LDL cholesterol levels were assessed with the HiCo Gen.2 assay, comprising 2100 tests on the cobas C platform (Roche Diagnostics, reference number 5168538190). Additionally, HDL cholesterol levels were measured using the Cobas C-Col HDL rct 500d assay (Roche Diagnostics, reference number 07528582190).

### 2.1 Surgical procedures

mRYGB combined restriction, creating a small gastric pouch of 100 ml, with hypo absorption that was accomplished by a 200 cm biliopancreatic limb and an alimentary limb of 100 cm.

### 2.2 T2D Remission

After one year of follow-up, complete remission was defined as an HbA_1c <_6% in the absence of hypoglycemic treatment (24). To consider that the patients achieved complete remission, we verified that they had an HbA1c <6% one year after surgery and without hypoglycemic treatment for at least 3 months previous to follow-up evaluation.

### 2.3 Stool sample collection, DNA extraction, and metagenomic sequencing

To assess taxonomic and functional changes in fecal samples collected we used shotgun sequencing of stool DNA for whole metagenome analysis. Patients collected their fresh stool samples at home, which were then immediately frozen in their home freezer at -20°C. Frozen samples were delivered to the hospital within two days using insulating polystyrene foam containers and were kept at -80°C until analysis. DNA extraction was performed using the QIAamp DNA stool kit (Qiagen, Hilden, Germany). DNA quantification was performed using a Qubit 3.0 Fluorometer (Thermo Fisher Scientific, Carlsbad, CA), and one ng of each sample (0.2 ng/μL) was used for shotgun library preparation using the Nextera XT DNA Library Preparation Kit (Illumina, Inc., San Diego, CA). Sequencing was carried out on a NextSeq 500 sequencer (Illumina) with 150-bp paired-end chemistry at the Sequencing and Bioinformatic Service of FISABIO (Valencia, Spain).

Taxonomic assignments of total DNA metagenomic sequencing were carried out through Kaiju (25), a program for computationally efficient and sensitive taxonomic classification of high-throughput sequencing reads from metagenomics sequencing experiments, by which each sequencing read is assigned to a taxon in the NCBI taxonomy (https://www.ncbi.nlm.nih.gov/taxonomy) by comparing it to the microbial subset of the NCBI BLAST non-redundant protein database, not including fungi and microbial eukaryotes.

### 2.4 Statistical analysis of taxonomical and clinical features

Sequence data were analyzed using the phyloseqR (version 1.28.0) (26), vegan (version 2.5-5) (27), metagenomeSeq (version 1.26.2) (28) and ggplot2 packages implemented in R. Taxonomical analysis reached species level if possible unless otherwise stated (-uc annotated).

Abundance raw-data counts were normalized using the cumulative sum scaling (CSS) method (28). The zero-inflated Gaussian mixture model (FitZig), was applied over the cumulative sum scaling-normalized data to account for abundance differences of species between pre-and post-treatment, by including the patient identifier variable (IDPAT) as a covariate in the analysis to account for the paired nature of the data. This approach has proven to be more effective than comparable differential abundance methods such as DESeq, edgeR or Voom (29).

To evaluate alpha diversity of bacterial communities, Shannon’s index and OTUs (Observed species) were calculated using the phyloseqR package. The proportion and composition of the most abundant species in the data was aggregated at both Phylum and Family levels. The beta diversity was computed under the Bray-Curtis dissimilarity index (30) and linked to clinical variables using the distance-based Adonis procedure (31). Implementation in the principal component analysis (PCA) on the CSS normalized data was applied to represent the percentage of explained data variation in the most relevant clinical variables, as well as to identify potential outliers and rare species. Clinical variables were tested for normality using the Shapiro-Wilk test before running inferential statistics. Non-parametric data were evaluated by the Wilcoxon rank-sum test, while normally distributed variables were examined by Student’s t-test. P-values less than 0.05 were considered significant after applying the Benjamini-Hochberg multiple testing procedure. The relationship between clinical variables and alpha diversity measures was evaluated using a linear mixed effect model, considering the alpha diversity as response variable, the clinical variable as fixed effect and the patient as random effect. Spearman’s rank correlation was used to investigate associations between microbial data and reported clinical variables using a customized z-score metric supported by a global signature correction approach (32) (33) (34).

Spearman’s rank correlation was also used to investigate associations between normalized microbial abundance counts, at a species level, with reported clinical variables. P-values were then adjusted with the false rate discovery method. Correlations and adjusted P-values were computed with R package stats (version 4.0.5).

### 2.5 Statistical analysis

Data are presented as mean ( ± SD) or percentage for normally distributed quantitative variables or median and interquartile range for non-normally distributed quantitative variables. The categorical variables were described as the number of cases and the percentage concerning the total. When necessary, the correlation between quantitative variables was calculated using Pearson’s or Spearman’s test. Paired t-tests and Wilcoxon signed-rank tests were used to evaluate the impact between groups according to each metabolic distribution. The area under the time concentration curve (AUC) for GLP-1, GLP-2, insulin, and glucose, was calculated using the trapezoidal rule (35).

Logistic regression analysis was used to determine variables associated with T2D remission. The model included the following variables: initial C-peptide levels, HbA_1c_, succinate concentrations, time of T2D duration, insulin treatment, total weight loss, and GLP-1 and 2 responses. Spearman’s rank correlation was used to investigate associations between normalized microbial abundance counts, at a species level, with reported clinical variables. *P*-values were then adjusted with the false rate discovery method. Correlations and adjusted *P*-values were computed with R package stats (version 4.0.5).

## 3 Results

From June 2016 to June 2017, 15 patients with severe obesity and T2D were consecutively recruited and included in the study; two could not complete the follow-up due to sudden death (n=1) and severe pneumonia with prolonged hospitalization in another patient (n=1). Finally, 13 patients were included in the analysis. The studied cohort had a mean age of 52.6 ± 6.5 years, a mean BMI of 39.3 ± 1.4 kg/m^2^, and an initial HbA_1c_ of 7.62 ± 1.5%, with 69.2% of patients under insulin treatment. **Table 1** summarizes the characteristics of the participants.

**Table 1:** Participant characteristics at baseline and 1 year post-surgery.

	Pre-surgery	Post-surgery	*P* overall
	N=13	N=13	
**Sex (male/female)**	4/9	4/9	
**Age (years)**	52.6 ±6.56	53.6 ±6.56	
**Weight (kg)**	103 ±13.2	68.1 ±14.2	<0.001
**BMI (kg/m²)**	39.3 ±1.44	25.8 ±2.08	<0.001
**Waist (cm)**	125 ±16.4	92.4 ±11.3	<0.001
**Hip (cm)**	125 ±13.1	100 ±6.62	<0.001
**Fat Mass (%)**	31.5 ±13.6	20.1 ±4.95	0.01
**Lean Mass (%)**	40.7 ±18.7	46.2 ±10.9	0.37
**Body Fat (%)**	43.1 ±5.30	29.6 ±6.13	<0.001
**Insulin treatment, N (%):**			
**Yes (%)**	9 (69.2)	1 (7.69)	
**No (%)**	4 (30.8)	12 (92.3)	
**FPG (mmol/l)**	9.9 ±4.6	5.29 ±1.4	0.03
**HbA_1c_ (%)**	7.62 ±1.5	5.44 ±0.85	<0.001
**HOMA-IR**	8.87 ±6.45	1.50 ±0.75	<0.001
**Total Cholesterol (mmol/L)**	4.90 ±1.01	4.07 ±0.66	0.02
**HDL (mmol/l)**	1.18 ±0.43	1.28 ±0.25	0.50
**LDL (mmol/l)**	2.93 ±0.87	2.30 ±0.6	0.08
**Triglycerides (mmol/L)**	2.93 ±2.97	1.25 ±0.58	0.07
**IL-6 (pg/mL)**	3.49 ±1.74	2.49 ±2.01	0.20
**Zonulin (ng/mL)**	3.13 ±0.45	2.49 ±0.41	<0.001
**Succinate (uMol)**	79.74 ±28.8	50.97 ±15.3	<0.001

Data are expressed as mean ± SD. P values were calculated using paired t-test. Fat, lean and body mass were measured by DEXA. BMI, Body mass index; FPG, Fasting plasma glucose; HOMA-IR, Homeostatic model assessment of insulin resistance; IL-6, Interleukin 6.

### 3.1 Metabolic profile changes after surgery

Twelve months after mRYGB, a dramatic reduction was observed in weight with 34.2 ± 6.1% of total weight loss (TWL) at the expense of a fat mass of 36.1 ± 5.1%. As expected, a significant metabolic improvement after surgery was found with a decrease in fasting plasma glucose, HOMA-IR, HbA1c levels, and lipid profile (**Table 1**). T2D remission was achieved in 69% of patients.

**Figure 1:**
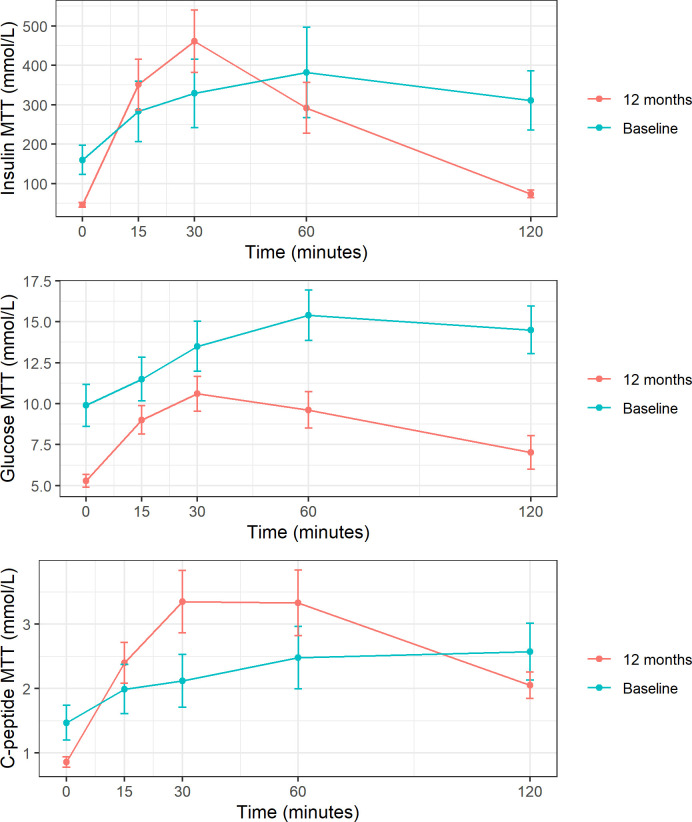
Insulin, glucose, and C-peptide response to MTT at baseline and 1 year post-surgery. These figures show mean and standard deviation of insulin, glucose and C-peptid (expressed in mmol/L) at each time point after meal tolerance test (MTT). Baseline represented in blue and follow-up in red.

Following MTT, AUC for glucose significantly decreased after the intervention (pre 1677 vs post 1049 mmol/L, *P*<0.05), whereas an increase in C-peptide AUC was observed (pre 277 vs post 325 mmol/L, *P*<0.001) (**Figure 1**). Postprandial serum C-peptide to plasma glucose concentration ratio significantly increased after surgery 0.24 ± 0.27 vs 0.98 ± 0.91, *P <*0.05.

As markers of systemic inflammation, IL-6 showed a trend to decrease after surgery, but without reaching statistical significance, and fasting plasma succinate was significantly reduced after surgery at 79.74 ± 28.8 vs. 50.97 ± 15.3 µmol/L, *P*=0.001.

### 3.2 Evaluation of intestinal permeability

Zonulin plasmatic levels are a reliable biomarker of the intestinal barrier integrity of the small intestine (36). It circulates in the blood and binds to a receptor in the enterocytes leading to dysfunction of tight junctions that finally increases small intestine permeability (37)

Zonulin significantly decreased after mRYGB 3.13 ± 0.45 vs 2.49 ± 0.41 ng/mL, (*P*=0.01).

### 3.3 Incretin profile changes with surgery

**Figure 2:**
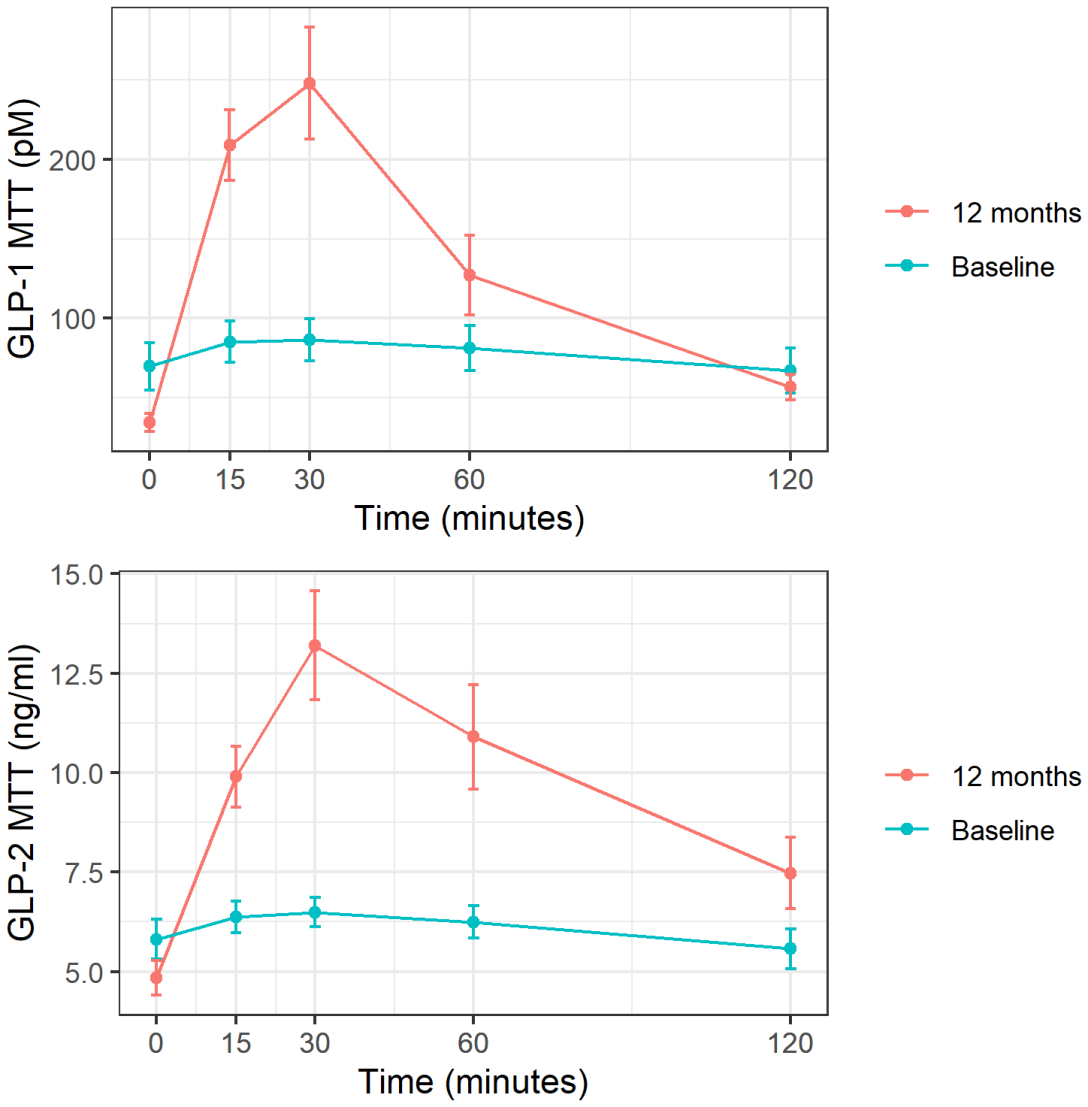
GLP-1 and GLP-2 response to MTT at baseline and 1 year post-surgery. These figures show mean and standard deviation of GLP-1 (expressed in pM) and GLP-2 (expressed in ng/ml) at each time point after meal tolerance test (MTT). Baseline represented in blue and follow-up in red.

When analyzing the incretin profile after MTT, a restoration of the typical slope for GLP-1 and GLP-2 was observed with the corresponding increase in AUC for both hormones after surgery: GLP-1 AUC pre 9731 vs post 15788 pM, P<0.05 and GLP-2 AUC pre 732 vs post 1190 ng/ml, P<0.001 (**Figure 2**). The same behavior was found after LT for both GLP-1 (AUC pre 13751 vs. post 21070 pM, *P*=0.01) and GLP-2 (AUC pre-1160 vs. post-1654 ng/ml, *P*=0.01).

Within the study sample comprising 13 participants, of which 9 were female, representing over 50% of the cohort, an investigation into potential differences in GLP-1 and GLP-2 levels before and after surgical intervention was conducted among the female subgroup, consisting of 4 pre-menopausal and 5 post-menopausal individuals. The analysis revealed that there were no statistically significant distinctions in GLP-1 and GLP-2 levels between the pre-menopausal and post-menopausal groups before and after the surgical procedure.

### 3.4 Changes in gut microbiota and relationship with anthropometric parameters

The surgical intervention had no significant impact on richness and evenness, measured by the Shannon diversity index. Nonetheless, it affected beta diversity (*P*=0.005) estimated by the Bray distance as a metric to describe overall microbiota structure. A common feature of microbiome data analysis was the statistical sparsity and the lack of homogeneously distributed variables among individuals. This limitation was overcome by applying the FitZig mixture model (28). This analysis of the elapsed studied time revealed that 111 different species significantly increased (*P* adj<0.05) after the follow-up, and 67 other species reduced considerably (*P* adj<0.05) in abundance after the intervention.

**Figure 3:**
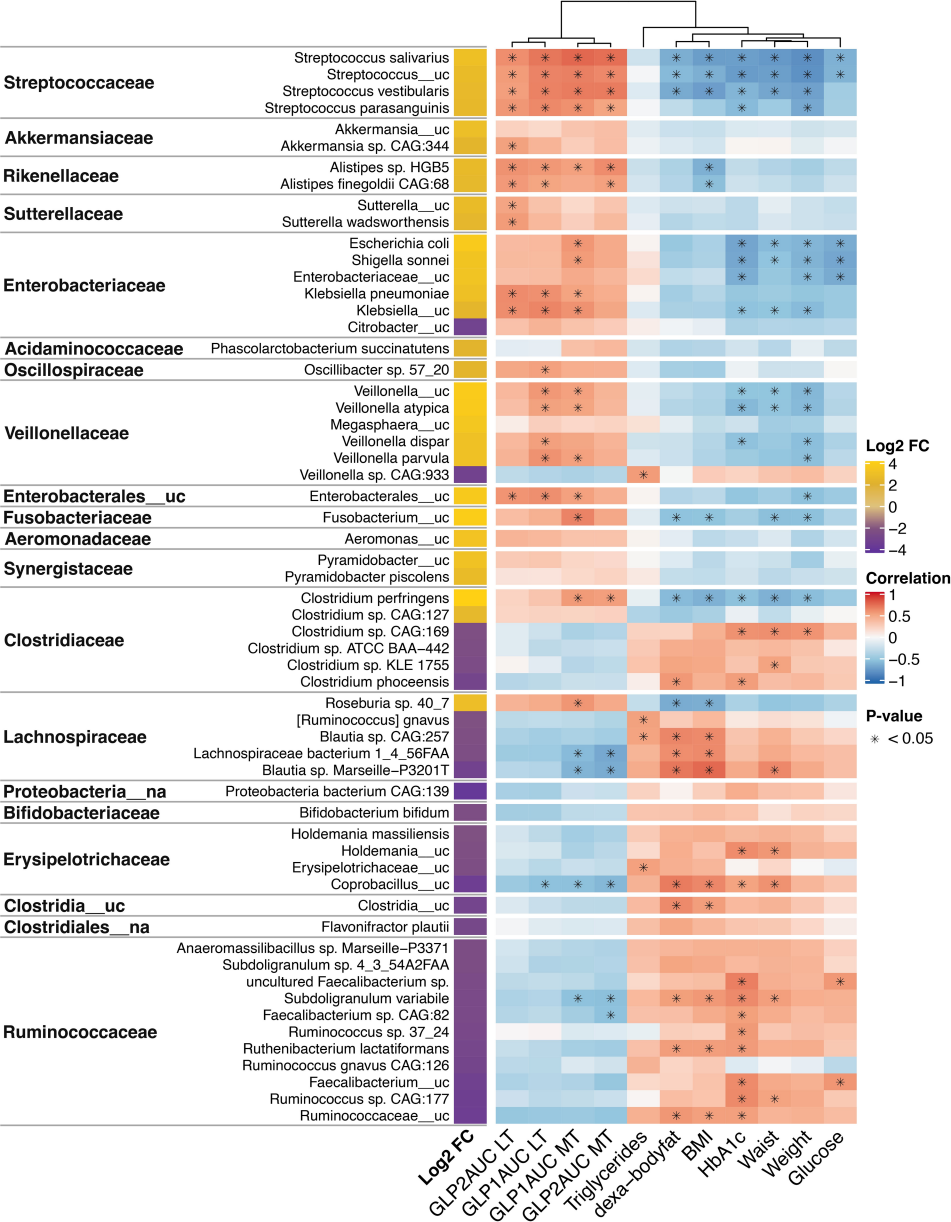
Changes and associations between species and metabolic characteristics 1 year post-surgery. Log2 fold-change (FC) expresses the significant increase or decrease (≥+2/-2, *P* adjusted <0.001) of microbiota species 1 year after surgery compared to baseline levels. It is based on the fitZig model. The yellow color denotes increase, and purple denotes decrease. The correlations of the species and metabolic variables are showed in a heatmap, where red denotes a positive correlation and blue a negative correlation. The intensity of the color is related to the strength of the correlation. Statistically significant associations (*P* adjusted <0.05) are marked with *. GLP1, glucagon-like peptide 1; GLP2, glucagon-like peptide 2; AUC, area under curve; MTT, Meal tolerance test; LT, lipid test. BMI, body mass index.

Most of the significantly increased taxa after the follow-up belonged to the Streptococcaceae (*Streptococcus salivarius*, *Streptococcus*_uc., *Streptococcus vestibularis* and S*treptococcus parasanguinis*), Akkermansiaceae (*Akkermansia* sp. CAG:344), Rickenellaceae (*Alistipes* sp. HGB5 and *Alistipes finegoldii* CAG:68), Sutterellaceae (*Sutterella*_uc and *Sutterella wadsworthensis*), Enterobacteriaceae (*Escherichia coli, Shigella sonnei, Klebsiella pneumoniae*, and *Klebsiella*_uc.), *Oscillospiraceae* (*Oscillibacter* sp. 57_20), Veillonellaceae (*Veillonella* _uc, *Veillonella atypica, Veillonella dispar and Veillonella parvula*), Enterobacterales_uc, and Fusobacteriaceae (*Fusobacterium*_uc) families. They showed a significant negative correlation (*P* adj<0.05) with some clinical and metabolic parameters studied (weight, BMI, waist circumference, body fat, HbA_1c_, glucose and triglycerides). (**Figure 3**)

Of note, *Clostridium perfringens* (Clostridiaceae family) and *Roseburia* sp. *40_7* (Lachnospiraceae family) that increased after follow-up showed a significant negative correlation with body fat and BMI, even belonging to families that generally decreased after the intervention and had opposite correlations.

On the other hand, downregulated species after surgery belong to the Clostridiaceae family (*Clostridium* sp. CAG:169, *Clostridium* sp. KLE 1755, and *Clostridium phoceensis)*, Lachnospiraceae family *(Blautia* sp. CAG257, *Lachnospiraceae bacterium* 1_4_56FAA, *Blautia Marseille-P3201T*), Erysipelotricaceae (*Holdemania*_uc and *Coprobaccillus*_uc), Clostridia_uc, and Rumnicocaceae (uncultured *Faecalibacterium* sp., *Subdoligranulum variabile*, *Faecalibacterium* sp. CAG:82, *Ruminococcus* sp. 37_24, *Ruthenibacterium lactatiformans*, *Faecalibacterium*_uc*, Ruminococcus* sp. CAG:177 and *Rumnicoccaceae_*uc) showed a significant positive correlation with some clinical and metabolic parameters reported (**Figure 3**).

We also analyzed the correlation of microbiota families with other variables, such as gut permeability markers (zonulin) but found no association. Of note, pre-surgical zonulin correlated positively with weight (r=0.61, *P*=0.027), and with pre-surgical AUC glucose (r=0.750, *P*=0.03).

### 3.5 Gut microbiota and incretin response

A close relationship between gut microbiota and incretin response after BS was found in our study. Most of the species that increased after BS that were previously mentioned belonged to the Streptococcaceae (*Streptococcus salivarius*, *Streptococcus*_uc., *Streptococcus vestibularis* and S*treptococcus parasanguinis*), Akkermansiaceae (*Akkermansia* sp. CAG:344), Rickenellaceae (*Alistipes* sp. HGB5 and *Alistipes finegoldii* CAG:68), Sutterellaceae (*Sutterella*_uc and *Sutterella wadsworthensis*), Enterobacteriaceae (*Escherichia coli, Shigella sonnei, Klebsiella pneumoniae*, and *Klebsiella*_uc.), Oscillospiraceae (*Oscillibacter* sp. 57_20), Veillonellaceae (*Veillonella* _uc, *Veillonella atypical, Veillonella dispar and Veillonella parvula*), Enterobacterales_uc, and Fusobacteriaceae (*Fusobacterium*_uc) families, revealed a significant positive correlation (*P* adj<0.05) with AUC of GLP-1 and/or GLP-2 after MTT and LT. Moreover, *Clostridium perfringens* (Clostridiaceae family) and *Roseburia* sp. *40_7* (Lachnospiraceae family) that increased after follow-up showed a positive correlation with AUC GLP-1 during the MTT. (**Figure 3** *).*

By contrast, some species that were downregulated after surgery belong to Lachnospiraceae (*Lachnospiraceae* bacterium 1_4_56FAA, *Blautia* sp. Marseille-P3201T), Erysipelotricaceae *(Coprobaccillus*_uc), Rumnicocaceae (*Subdoligranulum variabile* and *Faecalibacterium* sp. CAG:82) families, and showed a significant negative correlation with AUC for GLP-1 and/or GLP-2 after MTT and LT (**Figure 3** *)*.

### 3.6 T2D remission

Patients achieving complete T2D remission (69% of the sample) had higher initial C-peptide 1.83 ± 0.95 vs 0.67 ± 0.26 nmol/L (*P*=0.040), higher postprandial serum C-peptide to plasma glucose concentration ratio 1.28 ± 0.93 vs 0.30 ± 0.22 (*P*=0.015), lower T2D duration 9.6 ± 8.3 vs 16.2 ± 8.4 years (*P*=0.216), but similar proportion of insulin treatment compared to non-remitters. Higher pre-surgical and post-surgical AUC for C-peptide and insulin after MTT were observed in patients achieving T2D remission. However, AUC for GLP-1 and GLP-2 before and after surgery were similar, independently of metabolic outcomes.

In the multiple regression analysis, only higher initial C-peptide levels predicted better metabolic outcomes (R^2 = ^0.331, *P*=0.040), whereas pre-surgery HbA_1c,_ TWL, AUC for GLP-1, GLP-2, or succinate were not found to be determinants of T2D remission. Despite the association of the above species with some metabolic parameters, no specific species were linked to T2D remission.

## 4 Discussion

This study points to an association between a specific microbiome signature with a restoration of the incretin response, and metabolic improvement after mRYGB in patients with T2D, including a new player in post-surgery diabetes remission.

### 4.1 Association between gut microbiota, incretin response and metabolic parameters

A significant increase in GLP-1 and GLP-2 secretion during the MTT and LT after mRYGB was associated with changes in the microbiome.

To our knowledge, no previous study has analyzed the relationship between incretin response and the gut microbiota profile after metabolic surgery in patients with severe obesity and T2D.

In our cohort, a greater presence after surgery of species belonging to the families of Streptococcaceae, Akkermansiaceae, Rickenellaceae, Enterobacteriaceae, Oscillospiraceae, Veillonellaceae, Enterobacterales_uc, and Fusobacteriaceae, typically observed after massive weight loss in these patients, was associated with the improvement in the incretin (AUC for GLP-1 and GLP-2) and metabolic profile. Interestingly, species belonging to Ruminococcaceae, Erysipelotrichaceae, and Lachnospiraceae families that decreased after surgery showed an inverse association with the incretin response after an oral stimulus and with metabolic parameters. In contrast to other of their family species, *Clostridium perfringens* and *Roseburia* sp. 40_7 increased after surgery and correlated inversely with adiposity parameters and positively with incretin response.

Earlier studies have shown that BS induces a favorable shift into a healthier microbiome profile characterized by increased microbial richness (38) (39), and the changes are greater in RYGB compared to sleeve gastrectomy (SG) (40) (12). In our study, the overall changes after mRYGB in gut microbiota are similar to those previously described (40) (12) (41) (42) (43) (44) (45). However, with current evidence, it is difficult to associate the Firmicutes/Bacteroidetes ratio with a determined health status, including obesity (46). Our results showed that the species that decreased after surgery all belonged to the phylum Firmicutes, but the species that increased were heterogeneous, belonging to both the Firmicutes and Bacteroidetes phyla, as well as others.

These modifications can be explained by several factors other than diet modifications, such as the gastrointestinal tract’s rearrangement, a relevant anatomical shift, a determinant in bile acids production, and luminal pH changes (47). The lower gastric acid exposure might promote the proliferation of phylotypes from the oral cavity, such as *Escherichia*, *Veillonella*, *Streptococcus* genus (48), and also *Akkermansia*, which grows in a higher pH than the gastric microbes (49). Of note, these bacteria can ferment amino acids and carbohydrates into metabolites such as propionate and butyrate which have been associated with weight reduction (50), reduced gut permeability (51) (52) (53) (54) and a beneficial metabolic profile (55) (56), in line with the findings described in our study. On the other hand, we observed a decrease in species belonging to the families of Ruminococcaceae, Erysipelotrichaceae, and Lachnospiraceae, which belong to the Firmicute sphylum. As a phylum, Firmicutes are more acid adaptive and the increased alkaline environment following mRYGB is a factor along with a diet that might explain their reduction (57). As in other studies, we observed a decrease in the *Clostridium* species (with the exception of *Clostridium perfringens*), which could be partly explained because bypassing the duodenum introduces some oxygen to the gastrointestinal tract, inhibiting the growth of obligate anaerobes (58). However, changes in bile acids specifically the lower levels of all primary and secondary conjugated bile acids in the colon content after RYGBP has been linked in rodents with higher relative abundances of *Clostridium perfringens*, in agreement with our findings (59).

One proposed mechanism by which gut microbiota can influence metabolic outcomes is its potential ability to modify incretin secretion, among other gastrointestinal hormones (60). In rodent studies, it has been reported that healthier intestinal microbiome can provide increased luminal-derived secondary bile acids and propionate, activating the L-cell secretion of GLP-1 and GLP-2, enhancing insulin secretion and maintaining gut barrier integrity, respectively (61) (62). Also in mice, the use of probiotics, mainly *Lactobacillus*, has been associated with an enhancement of GLP-1 response and improvement of metabolic parameters (63). However, to date, there is scarce information linking specific bacteria with incretin dynamics after BS in animal models (57) (64) or humans (65). In other clinical settings, such as in healthy, normal weight subjects fed with resistant starch, a high baseline abundance of *Streptococcus* has been associated with increased postprandial levels of GLP-1, insulin, and C-peptide (66). Accordingly in our study, some species of Streptococcaceae family increased after surgery, and showed a significant positive association with AUC GLP-1 and GLP-2 after the MTT. Furthermore, in our study, increased GLP-2 secretion after an oral lipid load was also associated with some Akkermansiaceae species, and strikingly, GLP-1 secretion was also associated with the Veillonellaceae family and *Clostridium perfringens*. Although we cannot assume causality in these associations, some *in vivo* studies in mice and *in vitro* using human L-cell found that *Akkermansia muciniphila* stimulated GLP-1 by the secretion of a protein named P9, which binds to ICAM-2 receptors in L-cell (67). In rats undergoing RYGB, this bacterium was positively related to GLP-1 levels (64). Regarding Veillonellaceae, no previous direct association with incretins has been previously described. However, in a study performed on patients with non-alcoholic fatty liver treated with a fibroblast growth factor-19 analog, which decreased toxic bile acids, *Veillonella* was the only taxa exhibiting a significant increase (68). Therefore, as *Veillonella* seems to be a bile acid-sensitive bacterium, and these are known triggers of GLP-1 secretion, the association found in our study is reasonable and may be mediated by bile acid changes after BS. Commensal Clostridia are strongly involved in maintaining the overall gut function; in a previous study, *Clostridium asparagiforme* and *Clostridiales* bacterium *1_7_47FAA* increased and were positively correlated with postprandial GLP-1 in patients with obesity after SG (65). Another study increasing *Clostridium* sp. CAG:127 has been associated with GLP-2 response in subjects without severe obesity after diet-induced weight loss (21). Nevertheless, our finding of *Clostridium perfringens* association with GLP-1 has never been reported and requires further analysis as it may reflect potential beneficial effects of this species in the context of a hypoabsorptive technique. It is important to highlight that although *C. perfringens* can be a potential pathogen, it is a ubiquitous bacterium and part of the ecological community in the intestinal tract of humans (69).

Our data revealed that a better metabolic and incretin profile after surgery was linked to a specific gut microbiota composition. Still, the study failed to characterize a particular gut microbiota related to T2D remission, probably because it was underpowered to reach significance. Previous studies have described an increase in *Roseburia intestinalis* in patients achieving remission after RYGB and SG (20) (19), and *Akkermansia muciniphila* has been linked to a better metabolic profile mainly after SG (40) (70). As previously exposed, we observed an increase in *Roseburia* sp. and Akkermansiaceae family that was positively related to an enhanced incretin response and inversely with adverse adiposity and metabolic parameters.

It is important to take into account that there are numerous interspecies and inter-gut section interactions that profiles microbial functionality.

### 4.2 Gut permeability

Although microbiota has been linked to a reduction in gut permeability, we were unable to find an association of plasma zonulin with the gut microbiome studied.

### 4.3 T2D remission predictors

Some predictive pre-surgical factors of metabolic outcomes after BS have been identified, such as younger age, shorter disease duration, preoperative C-peptide levels, and the absence of insulin treatment before surgery, which are all associated with higher remission rates (21) (64) (71) (72) (73). C-peptide levels were the only significant predictor of T2D remission in our study.

### 4.4 Limitations

We acknowledge several limitations of this study. Sample size limitations can have influenced the statistical power to detect changes in T2D remission after surgery. Moreover, we were unable to study the composition of the diet followed by the participants after surgery, which may affect the results. We have also observed a high interindividual variation in gut microbial composition, which makes the analysis between metabolic variables and microbiome difficult. We are aware of the absence of causality in the findings described in our study. For these reasons, this study should be considered as a hypothesis generator. Further studies should therefore be conducted to better understand the changes after BS, especially in microbiota composition, and its association with metabolic outcomes.

## 5 Conclusions

Patients with obesity and T2D submitted to metabolic surgery by mRYGB improve their metabolic phenotype in parallel with significant modifications in the microbiome composition. Incretin response is restored after weight loss and is associated with a specific microbiota signature after one year of follow-up. These changes operate in parallel with T2D remission. Further studies are guaranteed to confirm a causal role of the microbiome changes on incretin response and T2D remission in patients with obesity.

## Data availability statement

The data presented in the study are deposited in the ENA website repository https://www.ebi.ac.uk/ena/submit/webin/, accession number PRJEB63100.

## Ethics statement

The studies involving humans were approved by Clinical Research Ethics Committee (reference PR 198/14). The studies were conducted in accordance with the local legislation and institutional requirements. The participants provided their written informed consent to participate in this study.

## Author contributions

LH-M, M-MR-P, JV and NVil contributed in the conception of the work and wrote the manuscript. RP contributed in the data analysis. BA performed the bioinformatics and statistical analyses. FG-P, NVir and RLP participated in the study design. JO, RM, CL, CS, MP-M, MP-P, SP and SF-V critically revised the manuscript. All authors contributed to the article and approved the submitted version.

## References

[1] Khorgami Z Shoar S Saber AA Howard CA Danaei G Sclabas GM. Outcomes of bariatric surgery versus medical management for type 2 diabetes mellitus: a meta-analysis of randomized controlled trials. Obes Surg (2019) 29(3):964–74. doi: 10.1007/s11695-018-3552-x 30402804

[2] Jørgensen NB Dirksen C Bojsen-Møller KN Jacobsen SH Worm D Hansen DL. Exaggerated glucagon-like peptide 1 response is important for improved β-cell function and glucose tolerance after roux-en-Y gastric bypass in patients with type 2 diabetes. Diabetes (2013) 62(9):3044–52. doi: 10.2337/db13-0022 PMC3749359 23649520

[3] Guida C Stephen SD Watson M Dempster N Larraufie P Marjot T. PYY plays a key role in the resolution of diabetes following bariatric surgery in humans. EBioMedicine (2019) 40:67–76. doi: 10.1016/j.ebiom.2018.12.040 30639417 PMC6413583

[4] Wewer Albrechtsen NJ Hornburg D Albrechtsen R Svendsen B Toräng S Jepsen SL. Oxyntomodulin identified as a marker of type 2 diabetes and gastric bypass surgery by mass-spectrometry based profiling of human plasma. EBioMedicine [Internet]. (2016) 7:112–20. doi: 10.1016/j.ebiom.2016.03.034 PMC4909640 27322465

[5] Muñoz-Garach A Diaz-Perdigones C Tinahones FJ. Microbiota y diabetes mellitus tipo 2. Endocrinol y Nutr (2016) 63(10):560–8. doi: 10.1016/j.endonu.2016.07.008 27633134

[6] Haeusler RA Astiarraga B Camastra S Accili D Ferrannini E. Human insulin resistance is associated with increased plasma levels of 12a-hydroxylated bile acids. Diabetes (2013) 62(12):4184–91. doi: 10.2337/db13-0639 PMC3837033 23884887

[7] Xu G Song M. Recent advances in the mechanisms underlying the beneficial effects of bariatric and metabolic surgery. Surg Obes Relat Dis (2021) 17(1):231–8. doi: 10.1016/j.soard.2020.08.028 PMC7769897 33036939

[8] Batterham RL Cummings DE. Mechanisms of diabetes improvement following bariatric/metabolic surgery. Diabetes Care (2016) 39(6):893–901. doi: 10.2337/dc16-0145 27222547 PMC5864134

[9] Anhê FF Varin TV Schertzer JD Marette A. The gut microbiota as a mediator of metabolic benefits after bariatric surgery. Can J Diabetes [Internet]. (2017) 41(4):439–47. doi: 10.1016/j.jcjd.2017.02.002 28552651

[10] Allin KH Nielsen T Pedersen O. Mechanisms in endocrinology: Gut microbiota in patients with type 2 diabetes mellitus. Eur J Endocrinol (2015) 172(4):R167–77. doi: 10.1530/EJE-14-0874 25416725

[11] Yang JY Kweon MN. The gut microbiota: A key regulator of metabolic diseases. BMB Rep (2016) 49(10):536–41. doi: 10.5483/BMBRep.2016.49.10.144 PMC5227294 27530685

[12] Palmisano S Campisciano G Silvestri M Guerra M Giuricin M Casagranda B. Changes in gut microbiota composition after bariatric surgery: a new balance to decode. J Gastrointest Surg (2020) 24(8):1736–46. doi: 10.1007/s11605-019-04321-x 31388884

[13] Zhang H DiBaise JK Zuccolo A Kudrna D Braidotti M. Human gut microbiota in obesity and after gastric bypass. Proc Natl Acad Sci (2009) 106(7):2365–70. 10.1073/pnas.0812600106 PMC2629490 19164560

[14] Li JV Ashrafian H Bueter M Kinross J Sands C Le Roux CW. Metabolic surgery profoundly influences gut microbial - Host metabolic cross-talk. Gut (2011) 60(9):1214–23. doi: 10.1136/gut.2010.234708 PMC3677150 21572120

[15] Assal K Prifti E Belda E Sala P Cl K carlota DM. Gut microbiota profile of obese diabetic women submitted to roux-en-Y gastric bypass and its association with food intake and postoperative diabetes remission. Nutrients (2020) 12(278):1–18. doi: 10.3390/nu12020278 PMC7071117 31973130

[16] Liou AP Paziuk M Luevano JM Jr. Machineni S Turnbaugh PJ Kaplan LM. Conserved shifts in the gut microbiota due to gastric bypass reduce host weight and adiposity. Sci Transl Med [Internet] (2013) 27(5(178):178r41. doi: 10.1126/scitranslmed.3005687 PMC3652229 23536013

[17] Shao Y Ding R Xu B Hua R Shen Q He K. Alterations of gut microbiota after roux-en-Y gastric bypass and sleeve gastrectomy in sprague-dawley rats. Obes Surg (2017) 27(2):295–302. doi: 10.1007/s11695-016-2297-7 27440168

[18] Lu C Li Y Li L Kong Y Shi T Xiao H. Alterations of serum uric acid level and gut microbiota after roux-en-Y gastric bypass and sleeve gastrectomy in a hyperuricemic rat model. Obes Surg (2020) 30(5):1799–807. doi: 10.1007/s11695-019-04328-y PMC7228899 32124218

[19] Murphy R Tsai P Jüllig M Liu A Plank L Booth M. Differential changes in gut microbiota after gastric bypass and sleeve gastrectomy bariatric surgery vary according to diabetes remission. Obes Surg (2017) 27(4):917–25. doi: 10.1007/s11695-016-2399-2 27738970

[20] Davies N O’Sullivan JM Plank LD Murphy R. Gut microbial predictors of type 2 diabetes remission following bariatric surgery. Obes Surg (2020) 30(9):3536–48. doi: 10.1007/s11695-020-04684-0 32447634

[21] Rodríguez-Peña M Astiarraga B Seco J Ceperuelo-Mallafré V Caballé A Pérez-Brocal V. Changes in glucagon-like peptide 1 and 2 levels in people with obesity after a diet-induced weight-loss intervention are related to a specific microbiota signature: A prospective cohort study. Clin Transl Med (2021) 11(11):1–7. doi: 10.1002/ctm2.575 34841718 PMC8571947

[22] Gomes-Rocha SR Costa-Pinho AM Pais-Neto CC Pereira A de A Martins Nogueiro JP Ramos Carneiro SP. Roux-en-Y gastric bypass vs sleeve gastrectomy in super obesity: a systematic review and meta-analysis. Obes Surg (2022) 32:170–85. doi: 10.1007/s11695-021-05745-8 34642872

[23] Mechanick JI Apovian C Brethauer S Garvey WT Joffe AM Kim J. Clinical practice guidelines for the perioperative nutrition, metabolic, and nonsurgical support of patients undergoing bariatric procedures – 2019 update. Surg Obes Relat Dis [Internet]. (2020) 16(2):175–247. doi: 10.1016/j.soard.2019.10.025 31917200

[24] Buse JB Caprio S Cefalu WT Ceriello A Del Prato S Inzucchi SE. How do we define cure of diabetes? Diabetes Care (2009) 32(11):2133–5. doi: 10.2337/dc09-9036 PMC2768219 19875608

[25] Menzel P Ng KL Krogh A. Fast and sensitive taxonomic classification for metagenomics with Kaiju. Nat Commun (2016) 7:11257. doi: 10.1038/ncomms11257 PMC4833860 27071849

[26] McMurdie PJ Holmes S. Phyloseq: an R package for reproducible interactive analysis and graphics of microbiome census data. PloS One (2013) 8(4):e61217. doi: 10.1371/journal.pone.0061217 PMC3632530 23630581

[27] Oksanen AJ Blanchet FG Friendly M Kindt R Legendre P Mcglinn D. Vegan. Encycl Food Agric Ethics. (2019), 2395–6. doi: 10.1007/978-94-024-1179-9_301576

[28] Paulson JN Colin Stine O Bravo HC Pop M. Differential abundance analysis for microbial marker-gene surveys. Nat Methods (2013) 10(12):1200–2. doi: 10.1038/nmeth.2658 PMC4010126 24076764

[29] Weiss S Xu ZZ Peddada S Amir A Bittinger K Gonzalez A. Normalization and microbial differential abundance strategies depend upon data characteristics. Microbiome (2017) 5(1):1–18. doi: 10.1186/s40168-017-0237-y 28253908 PMC5335496

[30] Bray JR Curtis JT. An ordination of the upland forest communities of southern wisconsin. Ecol Monogr (1957) 27(4):325–49. doi: 10.2307/1942268

[31] Anderson MJ. A new method for non-parametric multivariate analysis of variance. Austral Ecol (2001) 26(1):32–46. doi: 10.1111/j.1442-9993.2001.01070.pp.x

[32] Lee E Chuang HY Kim JW Ideker T Lee D. Inferring pathway activity toward precise disease classification. PloS Comput Biol (2008) 4(11):e1000217. doi: 10.1371/journal.pcbi.1000217 PMC2563693 18989396

[33] Efron B Tibshirani R. On testing the significance of sets of genes. Ann Appl Stat (2007) 1(1):107–29. doi: 10.1214/07-aoas101

[34] Mestres AC Llergo AB Attolini CSO. Adjusting for systematic technical biases in risk assessment of gene signatures in transcriptomic cancer cohorts. bioRxiv (2018). doi: 10.1101/360495

[35] Wolever TMS Jenkins DJA Jenkins AL Josse RG. The glycemic index: Methodology and clinical implications. Am J Clin Nutr (1991) 54(5):846–54. doi: 10.1093/ajcn/54.5.846 1951155

[36] Sturgeon C Fasano A. Zonulin, a regulator of epithelial and endothelial barrier functions, and its involvement in chronic inflammatory diseases. Tissue Barriers [Internet]. (2016) 4(4):1–19. doi: 10.1080/21688370.2016.1251384 PMC5214347 28123927

[37] Fasano A. Zonulin and its regulation of intestinal barrier function: The biological door to inflammation, autoimmunity, and cancer. Physiol Rev (2011) 91(1):151–75. doi: 10.1152/physrev.00003.2008 21248165

[38] Kong LC Tap J Aron-Wisnewsky J Pelloux V Basdevant A Bouillot JL. Gut microbiota after gastric bypass in human obesity: Increased richness and associations of bacterial genera with adipose tissue genes. Am J Clin Nutr (2013) 98(1):16–24. doi: 10.3945/ajcn.113.058743 23719559

[39] Chen G Zhuang J Cui Q Jiang S Tao W Chen W. Two bariatric surgical procedures differentially alter the intestinal microbiota in obesity patients. Obes Surg (2020) 30(6):2345–61. doi: 10.1007/s11695-020-04494-4 32152837

[40] Sánchez-Alcoholado L Gutiérrez-Repiso C Gómez-Pérez AM García-Fuentes E Tinahones FJ Moreno-Indias I. Gut microbiota adaptation after weight loss by Roux-en-Y gastric bypass or sleeve gastrectomy bariatric surgeries. Surg Obes Relat Dis [Internet]. (2019) 15(11):1888–95. doi: 10.1016/j.soard.2019.08.551 31648978

[41] Graessler J Qin Y Zhong H Zhang J Licinio J Wong M l. Metagenomic sequencing of the human gut microbiome before and after bariatric surgery in obese patients with type 2 diabetes: correlation with inflammatory and metabolic parameters. Pharmacogenomics J (2013) 13(6): 514–22. doi: 10.1038/tpj.2012.43 23032991

[42] Palleja A Kashani A Allin KH Nielsen T Zhang C Li Y. Roux-en-Y gastric bypass surgery of morbidly obese patients induces swift and persistent changes of the individual gut microbiota. Genome Med [Internet]. (2016) 8(1):67. doi: 10.1186/s13073-016-0312-1 PMC4908688 27306058

[43] Wang FG Bai RX Yan WM Yan M Dong LY Song MM. Differential composition of gut microbiota among healthy volunteers , morbidly obese patients and post − bariatric surgery patients. Exp Ther Med (2019) 17:2268–78. doi: 10.3892/etm.2019.7200 PMC6395995 30867711

[44] Shen N Ahlers M Patel K Gao Z Dutia R Blaser MJ. Longitudinal changes of microbiome composition and microbial metabolomics after surgical weight loss in individuals with obesity. Surg Obes Relat Dis Obes Relat Dis (2020) 15(8):1367–73. doi: 10.1016/j.soard.2019.05.038 PMC6722012 31296445

[45] Han Y Kim G Ahn E Jung S Jung Y Kim Y. Integrated metagenomics and metabolomics analysis illustrates the systemic impact of the gut microbiota on host metabolism after bariatric surgery. Diabetes Obes Metab (2022) 24(7): 1224–34. doi: 10.1111/dom.14689 PMC9313881 35257467

[46] Magne F Gotteland M Gauthier L Zazueta A Pesoa S Navarrete P. The firmicutes/bacteroidetes ratio: A relevant marker of gut dysbiosis in obese patients? Nutrients (2020) 12(5):1474. doi: 10.3390/nu12051474 PMC7285218 32438689

[47] Ilhan ZE Dibaise JK Isern NG Hoyt DW Marcus AK Kang DW. Distinctive microbiomes and metabolites linked with weight loss after gastric bypass, but not gastric banding. ISME J [Internet]. (2017) 11(9):2047–58. doi: 10.1038/ismej.2017.71 PMC5563958 28548658

[48] Morales-Marroquin E Hanson B Greathouse L de la Cruz-Munoz N Messiah SE. Comparison of methodological approaches to human gut microbiota changes in response to metabolic and bariatric surgery: A systematic review. Obes Rev (2020) 21(8):1–15. doi: 10.1111/obr.13025 32249534

[49] Derrien M Vaughan EE Plugge CM de Vos WM. Akkermansia municiphila gen. nov., sp. nov., a human intestinal mucin-degrading bacterium. Int J Syst Evol Microbiol (2004) 54(5):1469–76. doi: 10.1099/ijs.0.02873-0 15388697

[50] Lin HV Frassetto A Kowalik EJ Nawrocki AR Lu MM Kosinski JR. Butyrate and propionate protect against diet-induced obesity and regulate gut hormones via free fatty acid receptor 3-independent mechanisms. PloS One (2012) 7(4):1–9. doi: 10.1371/journal.pone.0035240 PMC3323649 22506074

[51] Fouladi F Carroll IM Sharpton TJ Bulik-Sullivan E Heinberg L Steffen KJ. A microbial signature following bariatric surgery is robustly consistent across multiple cohorts. Gut Microbes [Internet]. (2021) 13(1):1–16. doi: 10.1080/19490976.2021.1930872 PMC8224199 34159880

[52] Zheng L Kelly CJ Battista KD Schaefer R Lanis JM Alexeev EE. Microbial-derived butyrate promotes epithelial barrier function through IL-10 receptor-dependent repression of claudin-2. J Immunol (2017) 199(8):2976–84. doi: 10.4049/jimmunol.1700105 PMC5636678 28893958

[53] Lupien-Meilleur J Andrich DE Quinn S Micaelli-Baret C St-Amand R Roy D. Interplay between gut microbiota and gastrointestinal peptides: potential outcomes on the regulation of glucose control. Can J Diabetes [Internet]. (2020) 44(4):359–67. doi: 10.1016/j.jcjd.2019.10.006 32057671

[54] Chelakkot C Choi Y Kim DK Park HT Ghim J Kwon Y. Akkermansia muciniphila-derived extracellular vesicles influence gut permeability through the regulation of tight junctions. Exp Mol Med [Internet]. (2018) 50(2):e450–11. doi: 10.1038/emm.2017.282 PMC5903829 29472701

[55] Coppola S Avagliano C Calignano A Berni Canani R. The protective role of butyrate against obesity and obesity-related diseases. Molecules (2021) 26(3):682. doi: 10.3390/molecules26030682 PMC7865491 33525625

[56] Chambers ES Viardot A Psichas A Morrison DJ Murphy KG Zac-Varghese SEK. Effects of targeted delivery of propionate to the human colon on appetite regulation, body weight maintenance and adiposity in overweight adults. Gut (2015) 64(11):1744–54. doi: 10.1136/gutjnl-2014-307913 PMC4680171 25500202

[57] Dang JT Mocanu V Park H Laffin M Tran C Hotte N. Ileal microbial shifts after Roux-en-Y gastric bypass orchestrate changes in glucose metabolism through modulation of bile acids and L-cell adaptation. Sci Rep [Internet]. (2021) 11(1):1–11. doi: 10.1038/s41598-021-03396-4 34893681 PMC8664817

[58] Farin W Oñate FP Plassais J Bonny C Beglinger C Woelnerhanssen B. Impact of laparoscopic Roux-en-Y gastric bypass and sleeve gastrectomy on gut microbiota: a metagenomic comparative analysis. Surg Obes Relat Dis (2020) 16(7):852–62. doi: 10.1016/j.soard.2020.03.014 32360114

[59] Haange SB Jehmlich N Krügel U Hintschich C Wehrmann D Hankir M. Gastric bypass surgery in a rat model alters the community structure and functional composition of the intestinal microbiota independently of weight loss. Microbiome (2020) 8):1–17. doi: 10.1186/s40168-020-0788-1 32033593 PMC7007695

[60] Rastelli M Cani PD Knauf C. The gut microbiome influences host endocrine functions. Endocr Rev (2019) 40(5):1271–84. doi: 10.1210/er.2018-00280 31081896

[61] Cani PD Possemiers S Van De Wiele T Guiot Y Everard A Rottier O. Changes in gut microbiota control inflammation in obese mice through a mechanism involving GLP-2-driven improvement of gut permeability. Gut (2009) 58(8):1091–103. doi: 10.1136/gut.2008.165886 PMC2702831 19240062

[62] Drucker DJ Ehrlich P Asa SL Brubaker PL. Induction of intestinal epithelial proliferation by glucagon-like peptide 2. Proc Natl Acad Sci U S A. (1996) 93(15):7911–6. doi: 10.1073/pnas.93.15.7911 PMC38848 8755576

[63] Wang Y Dilidaxi D Wu Y Sailike J Sun X hua NX. Composite probiotics alleviate type 2 diabetes by regulating intestinal microbiota and inducing GLP-1 secretion in db/db mice. BioMed Pharmacother [Internet] (2020) 125:109914. doi: 10.1016/j.biopha.2020.109914 32035395

[64] Yan M Song MM Bai RX Cheng S Yan WM. Effect of Roux-en-Y gastric bypass surgery on intestinal Akkermansia muciniphila. World J Gastrointest Surg (2016) 8(4):301. doi: 10.4240/wjgs.v8.i4.301 27152136 PMC4840169

[65] Hong J Bo T Xi L Xu X He N Zhan Y. Reversal of functional brain activity related to gut microbiome and hormones after VSG surgery in patients with obesity. J Clin Endocrinol Metab (2021) 106(9):E3619–33. doi: 10.1210/clinem/dgab297 PMC8372652 33950216

[66] Zhang L Ouyang Y Li H Shen L Ni Y Fang Q. Metabolic phenotypes and the gut microbiota in response to dietary resistant starch type 2 in normal-weight subjects: a randomized crossover trial. Sci Rep (2019) 9(1):1–11. doi: 10.1038/s41598-018-38216-9 30894560 PMC6426958

[67] Yoon HS Cho CH Yun MS Jang SJ You HJ hyeong KJ. Akkermansia muciniphila secretes a glucagon-like peptide-1-inducing protein that improves glucose homeostasis and ameliorates metabolic disease in mice. Nat Microbiol (2021) 6(5):563–73. doi: 10.1038/s41564-021-00880-5 33820962

[68] Loomba R Ling L Dinh DM DePaoli AM Lieu HD Harrison SA. The commensal microbe veillonella as a marker for response to an FGF19 analog in NASH. Hepatology (2021) 73(1):126–43. doi: 10.1002/hep.31523 PMC7898628 32794259

[69] Sawires YS Songer JG. Clostridium perfringens: Insight into virulence evolution and population structure. Anaerobe (2006) 12(1):23–43. doi: 10.1016/j.anaerobe.2005.10.002 16701609

[70] Salles BIM Cioffi D Ferreira SRG. Probiotics supplementation and insulin resistance: a systematic review. Diabetol Metab Syndr (2020) 12(1):1–24. doi: 10.1186/s13098-020-00603-6 33292434 PMC7656736

[71] Hall TC Pellen MGC Sedman PC Jain PK. Preoperative factors predicting remission of type 2 diabetes mellitus after Roux-en-Y Gastric bypass surgery for obesity. Obes Surg (2010) 20(9):1245–50. doi: 10.1007/s11695-010-0198-8 20524158

[72] Hamza N Abbas MH Darwish A Shafeek Z New J Ammori BJ. Predictors of remission of type 2 diabetes mellitus after laparoscopic gastric banding and bypass. Surg Obes Relat Dis (2011) 7(6):691–6. doi: 10.1016/j.soard.2010.03.292 20688578

[73] Dixon JB Chuang LM Chong K Chen SC Lambert GW Straznicky NE. Predicting the glycemic response to gastric bypass surgery in patients with type 2 diabetes. Diabetes Care (2013) 36(1):20–6. doi: 10.2337/dc12-0779 PMC3526207 23033249

